# Azadirachtin-Based Biopesticide Affects Fitness and Ovarian Development of the Natural Enemy *Ceraeochrysa claveri* (Neuroptera: Chrysopidae)

**DOI:** 10.3390/plants14030416

**Published:** 2025-01-31

**Authors:** Bertha Gastelbondo-Pastrana, Marilucia Santorum, Elton Luiz Scudeler, Fábio Henrique Fernandes, Erasmo Manuel Alvis, Linda Chams-Chams, Daniela Carvalho dos Santos

**Affiliations:** 1 Laboratory of Insects, Department of Structural and Functional Biology, Institute of Biosciences of Botucatu, São Paulo State University (UNESP), Botucatu 05508-070, SP, Brazil; gastelbondo.pastrana@unesp.br (B.G.-P.); mari_santorum@hotmail.com (M.S.); elton.scudeler@unesp.br (E.L.S.); 2 Medical and Pharmaceutical Sciences Group, Department of Medicine, School of Health Sciences, University of Sucre, Sincelejo, Sucre 700003, Colombia; 3 Grupo de Investigaciones Microbiológicas y Biomédicas de Córdoba—GIMBIC, Universidad de Córdoba, Monteria 230001, Colombia; ealvis@correo.unicordoba.edu.co (E.M.A.); lmchams@correo.unicordoba.edu.co (L.C.-C.); 4 Laboratory of Toxicogenomic and Nutrigenomic, Department of Pathology, Medical School, São Paulo State University (UNESP), Botucatu 05508-070, SP, Brazil; fernandesfh@gmail.com; 5 Electron Microscopy Center, Institute of Biosciences of Botucatu, São Paulo State University (UNESP), Botucatu 05508-070, SP, Brazil

**Keywords:** ovary, non-target insect, developmental toxicity, natural compounds, green lacewing

## Abstract

Plant-derived biopesticides have gained attention in agriculture as a pest control method that minimizes the negative effects caused by conventional synthetic insecticides to natural enemies. Azamax™ is one of the most commercialized biopesticides in Brazil, but little is known about its effects on non-target insects such as *Ceraeochrysa claveri*, a non-target insect that is economically important as a pest predator, used in this study. To evaluate the toxic effects of azadirachtin on fitness and ovarian development, a total of 450 *C. claveri* larvae were exposed by ingestion to subdoses (36 mg/L (0.3%) and 60 mg/L (0.5%) of azadirachtin for 15 days and after that, biological parameters and ovarian development were analyzed. The doses tested corresponded to the minimum and maximum concentrations used in the field. The results demonstrated that both tested doses of the biopesticide significantly reduced survival rates, delayed and extended larval and pupal development times, caused malformations in the body, altered the ultrastructure of adult ovaries, and induced cell death in ovarian follicles. Azamax™, a biopesticide marketed as a reduced-risk insecticide, was shown to have detrimental effects on the lifespan and ovarian development of *C. claveri*.

## 1. Introduction

*Ceraeochrysa* species have been studied due to their current use in commercial agriculture [1]. Many of these species are distributed over a variety of ecosystems and have been reported in agroecosystems [2]. Among these species, the green lacewing *Ceraeochrysa claveri* (Neuroptera: Chrysopidae) (Navás, 1911) is considered a polyphagous predator with great potential for biological control in neotropical areas; this is due to its key effect as a natural enemy on several arthropod pests [2,3], and it is considered a suitable biological model in ecotoxicological studies to represent predator insects [4,5]. At the larval stage, *C. claveri* has a broad range of soft-bodied prey, such as mites, aphids, whiteflies, thrips, and eggs and larvae of economically relevant insect pests. For these reasons, *C. claveri* is recommended to be included in Integrated Pest Management (IPM) programs [1,2,3]. Preserving non-target beneficial insects like *C. claveri* in agroecosystems when using synthetic or botanical insecticides is important for sustainable, integrated pest management [6,7,8,9,10,11].

However, to verify the compatibility of conventional pesticides and biopesticides with beneficial insects, it is necessary to assess the side effects in these insects before their joint use [6,12]. The results of different studies could be used by agronomist and environmental agencies to choose safe products to use in IPM programs and to facilitate the registration of new enabled products in agriculture [7].

Among the reduced-risk products, the search or demand for natural compounds, especially those derived from plant extracts, is high. The biopesticides obtained from the Indian neem tree (*Azadirachta indica* A. Juss) (Meliaceae) are widely used in agricultural crops against arthropod pests [13,14,15]. The most promising and effective insecticide extracted from the neem tree is azadirachtin, a tetranortriterpenoid with different action mechanisms that affects arthropod biology mainly as a repellent, antifeedant, and insect growth inhibitor and interferes with the mating behavior, fecundity, and fertility of female arthropod pests with different feeding habits [11,16,17,18]. It combines antifeedant action [19] with growth regulation and sterilant effects, caused mainly by alterations of ecdysteroid and juvenile hormone titers [20]. Negative effects of extracts from the neem tree on reproduction have also been reported in other insects [21,22,23], and it is assumed that azadirachtin has direct effects on a variety of tissues and organs, revealing several pathways and modes of action [24,25]. Although previous studies have tended to focus on a single physiological and morphological effect of azadirachtin in insects, recent results have shown multiple toxicity effects of this product [26].

The increasing demand for these biopesticides has led to a strong interest in studying their potential to cause negative effects on non-target species. In addition to broad-spectrum and high-efficiency activity against arthropod pests, azadirachtin-based products may also cause deleterious effects on beneficial insects, such as *C. claveri* [4,27,28,29,30,31,32,33]. Many azadirachtin-based commercial products are commercially available for use throughout the world in agricultural crops. In Brazil, the biopesticide is available in an emulsifiable concentrate formulation termed Azamax™, and its use is authorized to control arthropod pests in different crops [34].

In the food production chain, before the large-scale application of a biopesticide for pest insect control, it is important to confirm its multiple sublethal effects on natural predator insects as part of an effective pesticide risk assessment to provide sustainability and economic integration [5,35,36]. In this sense, we evaluated the effects of this azadirachtin-based product on biological parameters and ovarian development in *C. claveri.* It is important to assess the harmful effects of biopesticides on natural enemies, and further, it is particularly necessary to evaluate whether the defects can be broadcast to subsequent generations. Thus, this study evaluated the effects on the animals directly affected by the biopesticide and their offspring.

## 2. Results

In this study, we examined the potential effects of Azamax™ on insect fitness and its developmental consequences, in addition to the development of ovarian follicles from females after indirect ingestion (prey poisoning) during the larval period of *C. claveri*. Our results show that mortality of the immature insect stage increases at both of the chosen doses of the biopesticide. Moreover, the prepupal and pupal stages represent the most sensitive parts of the life cycle of *C. claveri*, leading to a decrease in adult emergence.

### 2.1. Evaluation of Azamax™ Toxicity in Immature Insect Stages

In the larval period, accumulated mortality showed a significant increase at the third instar, as demonstrated in Table 1. Most of these larvae died just prior to molting into the prepupal stage (Table 2). At third instar, the percentage of mortality recorded in controls was only 6.8%. When the treated remaining larvae from the third instar tried to molt to the prepupal stage, few individuals were able to ecdyse. A high percentage of the larvae of the last instar were unable to undergo or terminate ecdysis at both treatment doses (0.3% and 0.5%).

**Figure 1:**
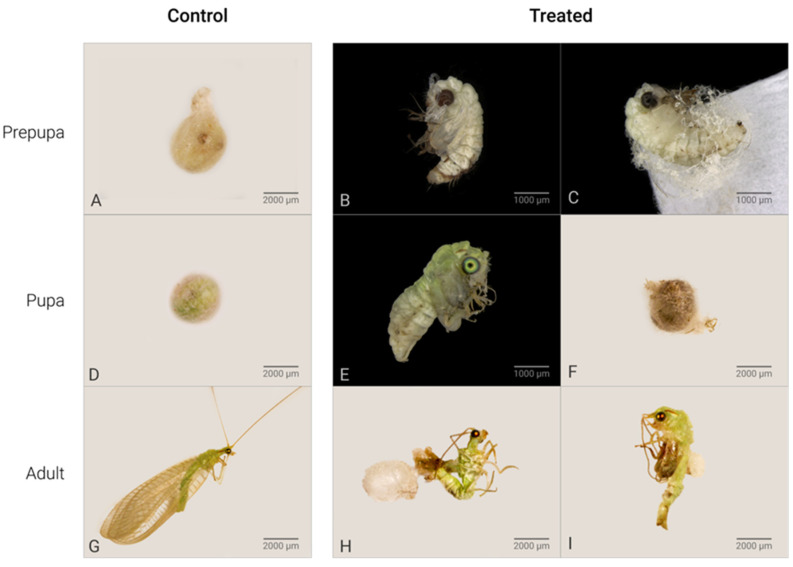
Effects of Azamax™ treatments (0.3 and 0.3%) on the lifecycle of *C. claveri* *. (**A**) Control prepupa: normal cocoon spun by an untreated larva; (**B**–**F**) alterations of Azamax™ treatments. (**B**) Malformed treated prepupa: exarate prepupa without cocoon; (**C**) exarate prepupa outside a malformed cocoon; (**D**) pupa: normal cocoon with a viable pupa inside; (**E**) malformed pupa: intermediate stage of development at which the pupa emerged with incomplete metamorphosis; (**F**) dark cocoon indicating pupal mortality inside the cocoon; (**G**) adult of control group: lateral view of a normal emerged adult; (**H**) malformed adults: malformed adult trying to emerge from a cocoon with altered tegument and deformed antennae, legs, and wings; (**I**) malformed adult at the intermediate stage of development that emerged after exposure during the larval period. * All effects were observed at both doses of Azamax™.

**Figure 2:**
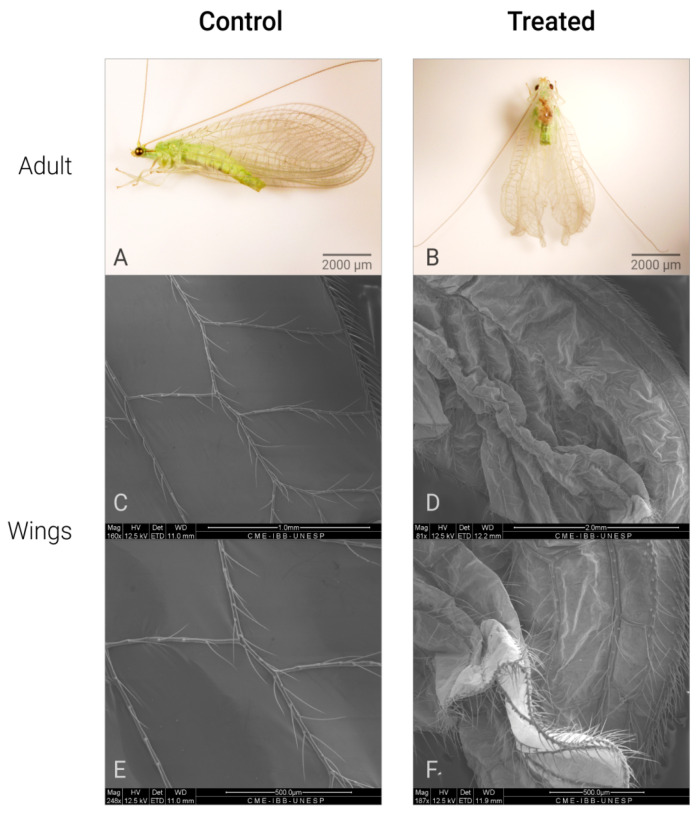
Different malformations of wings of *C. claveri* after exposure to Azamax™ treatments *. (**A**) Control adult: adult insect with normal wings; (**B**) treated adult (0.3 and 0.5% of Azamax™ group); adult insect with abnormal wings; (**C**,**E**) SEM micrographs of the normal wings; (**D**,**F**) SEM micrographs of the malformed wings. * All alterations were observed at both doses of Azamax™.

*C. claveri* has three larval instars, at the end of which the larva spins a silk cocoon [27]. After cocoon spinning by the prepupae, their cocoons have a round shape with a heterogeneous structure that consists of two distinct layers [27]. In this study, the cocoons of untreated larvae were regular with a rounded shape (Figure 1A). The main effect of Azamax™ on the treated larvae was the inability to spin a cocoon in this period. The evidence of the above was the presence of nonviable prepupae, such as deformed prepupae with incomplete cocoons or without cocoons, leading to death (Figure 1B,C,E,F). In addition, detailed changes in the morphology of the wings after exposure to Azamax™ are shown in Figure 2.

The quantitative effect of Azamax™ on prepupal survival is shown in Table 2, represented by the percentage of pupation (percentage of pupae formed). Significant differences were found in both treatments compared to the control, which achieved 92% pupation. The abilities of the treated larvae to molt into pupae for both the 0.3 and 0.5% treatments were 54.7% and 40%, respectively.

Negative effects of treatment were also visible at the pupal stage (Figure 1B,C). At the pupal stage, the organisms treated with Azamax™ showed differences in external morphology when compared to the control; the treated pupae were not able to complete ecdysis and metamorphosis inside of the cocoon (malformed pupae), while others died inside of the cocoon (Figure 1E,F). The malformed specimens at the end of pupal development presented characteristics of both pupae and adults, indicating unfinished metamorphosis.

Regarding cumulative mortality, represented by the percentage of individuals in the immature stages that did not reach the adult form, the results showed significant differences compared to the control (Table 2). Of the untreated larvae, 92% reached the adult stage, while in the group treated with a 0.5% dose of Azamax™, 64.7% of the insects failed to reach adulthood. As the Azamax™ dose increased, the emergence rate of adults decreased, as shown in Table 2. Consequently, this natural compound is considered highly toxic to *C. claveri* during the immature stages at both tested doses.

### 2.2. Effects on Developmental Time

**Figure 3:**
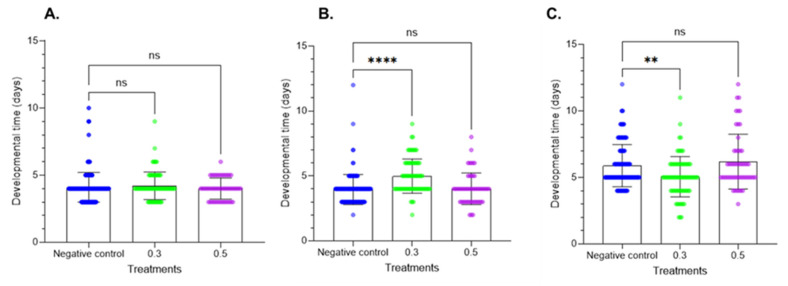
Effects of Azamax™ on larval development (mean ± SD) of the different larval stages of *C. claveri* when fed the eggs of *Diatraea saccharalis* treated with Azamax™. (**A**) First instar; (**B**) second instar; (**C**) third instar. Treatment groups: 0.3 and 0.5% of Azamax™, and negative control group treated with distilled water. ** Significant difference compared with the control group (*p* < 0.05; Kruskal–Wallis). **** Significant difference compared with the control group (*p* < 0.001; Kruskal–Wallis). ns: No significant difference compared with the control group (*p* < 0.05; Kruskal–Wallis).

**Figure 4:**
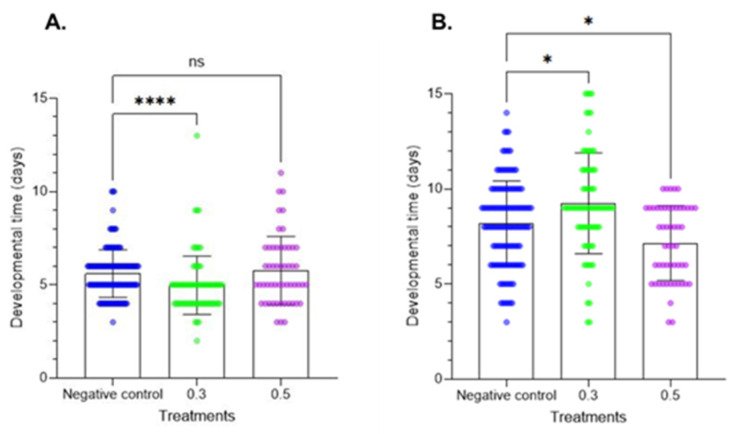
Developmental times of the prepupal and pupal stages (mean ± SD) of *C. claveri* treated with Azamax™ during larval development. (**A**) Prepupal stage; (**B**) pupal stage. Treatment groups: 0.3 and 0.5% of Azamax™, and negative control group treated with distilled water. * Significant difference compared with the control group (*p* < 0.05; Kruskal–Wallis). **** Significant difference compared with the control group (*p* < 0.001; Kruskal–Wallis). ns: No significant difference compared with the control group (*p* < 0.05; Kruskal–Wallis).

The larval development period data are provided in Figure 3. Treatment of fresh larvae altered the larval development period at the two tested doses. At the first instar, the results did not show significant differences. In contrast, at the second instar, Azamax™ at the minimal dose induced a delay in development, while the high dose had no effect on developmental time. In the prepupal stage, a decrease in the developmental time was observed at 0.3% Azamax™, but the same group of treated insects at the pupal stage showed a delay in the duration of the stage, as observed in Figure 4. The pupal period of the treated insects at 0.5% presented a reduction in the duration compared with the control insects.

#### 2.2.1. Effects of Azamax™ on the External Morphology of Ovarioles

**Figure 5:**
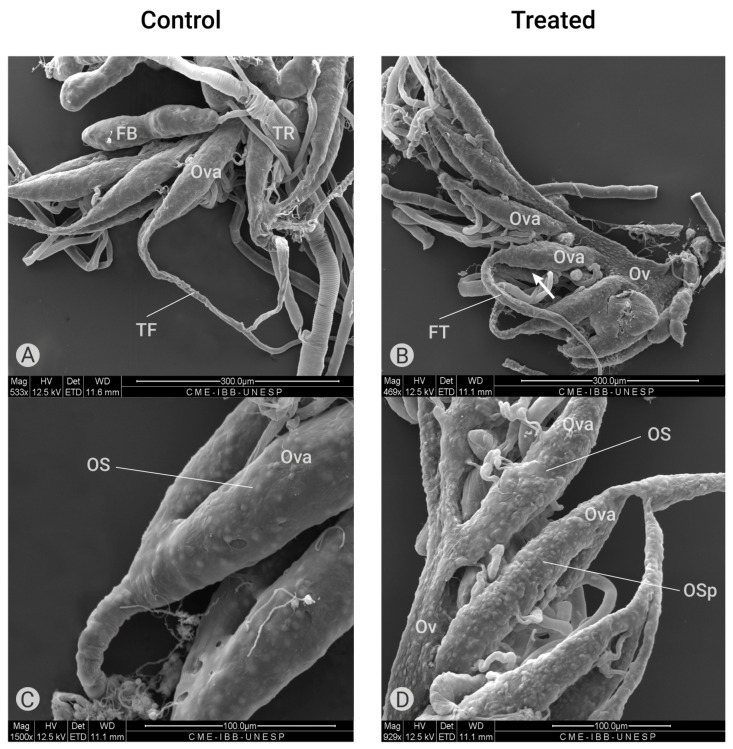
Ultrastructure of the ovary (scanning electron microscopy). (**A**) External morphology of ovaries of a control group containing ovarioles (Ova) with terminal filaments (TF) and fat bodies (FB). (**B**) External morphology of ovaries treated with Azamax (0.3% and 0.5%) showing the oviduct (Ov), shorter ovarioles (Ova) and a longer terminate filament (TF). (**C**) External superficies of ovarioles (Ova) of the control group showing the ovariole sheath (OS) with normal aspect. (**D**) External morphology of ovarioles treated with Azamax (0.5%) showing the ovariole sheath with irregular aspect containing protuberances (OSp).

This is the first report of the morphology (external and ultrastructure) of the ovary of *C. claveri*; therefore, the control group will be described in detail. The ovarioles of *C. claveri* are elongated and externally covered by a connective tissue sheath that contains a reticulum of muscle, the ovariole sheath (Figure 5A,C) [37].

According to Cruickshank [38], the ovariole sheath in insects, composed of a network of small and large cell layers, appears to be continuous over the ovarian follicle cells but discontinuous between these structures. This author observed that large pores were present in the sheath only between the oocyte chambers, while externally, large pores were observed between the strands of the sheath. In *C. claveri* ovaries, each pair of ovarioles is connected by a unique terminal filament (Figure 5A). In the group treated with Azamax (0.3% and 0.5%), the terminal filament is shorter in the proximal region to the oviduct and larger in size, as shown in Figure 5B. Additionally, we observed structures resembling large pores on the external surface of the ovariole sheaths in the treated ovarioles (Figure 5D).

#### 2.2.2. Effects of Azamax™ on Ovarian Follicle Organization

**Figure 6:**
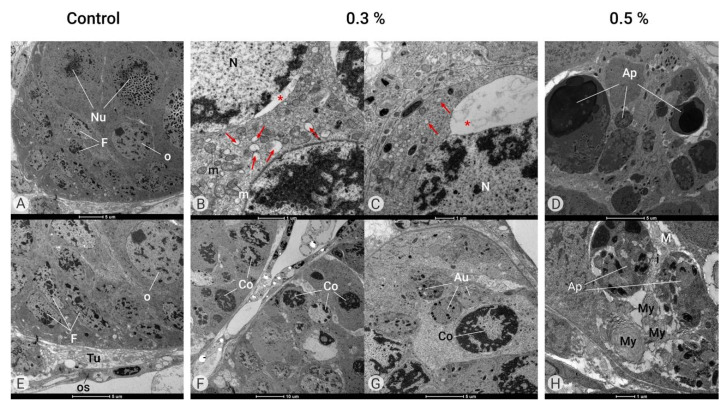
Ultrastructure of the ovary (TEM). Control Group (**A**,**E**). Ovaries of female adults at 1 day in age containing ovarian follicles composed of germ cells such as oocytes (O) and nurse cells (Nu) surrounded by follicular cells (**F**) and covered by tunica propria (Tu) and an opaque sheath (OS). Azamax™ treatment—0.3% (**B**,**C**,**F**,**G**). Note the large dilatation of the endoplasmic reticulum cisterns (arrows) and an important nuclear envelope dilatation (*) and condensed nucleus (Co) with normal mitochondria (m). Many autophagosomes (Au) have heterogeneous material and some organelles. Azamax™ treatment—0.5% (**D**,**H**). Note the many apoptotic bodies (Ap) and myelin figures (My), indicating the apoptotic cell death process.

The pair of ovaries of this insect are formed by polytrophic meroistic ovarioles, which contain ovarian follicles in different stages of development, similar to other insects in the family [37]. Ultrastructural analyses showed ovarian follicles composed of oocytes, nurse cells, and follicular cells in different stages of development. In the control group, we observed germ cells (oocyte and nurse cells) surrounded by follicle cells. The nuclei of the germ cells are round with regular contours and decondensed chromatin, and the follicle cells exhibit small nuclei with irregular contours (Figure 6A,E).

At both doses, Azamax™ treatment seems to be toxic to the adult ovaries, as the ultrastructure results revealed significant alterations in cellular organization into the ovaries (Figure 6). In the group treated with 0.3% Azamax™, it was possible to observe a large dilatation of the endoplasmic reticulum cisterns and an important nuclear envelope dilation (Figure 6B,C). Moreover, we also detected a condensed nucleus (Figure 6F,G). Many autophagosomes had heterogeneous material and some organelles (Figure 6G).

At 0.5% Azamax™, the intercellular spaces observed in the control group between somatic and germ cells and between adjacent germ cells seemed to have discrete enlargement. We also observed many apoptotic bodies and myelin figures, indicating the apoptotic cell death process (Figure 6D,H). Azamax™ seems to be more aggressive to the ovaries at the maximum dose, since the cellular alterations were more prominent in the 0.5% treatment group. However, these alterations suggested the absence of oocytes reaching the egg stage at both treatment doses.

## 3. Discussion

The fact that this biopesticide had an innocuous effect on the young larvae (first and second instar) could be explained by the shorter exposure time during this period, compared to when they reached the third instar. According to Garzón et al. [7], the higher cumulative larval mortality observed at the third instar was likely due to these organisms being more vulnerable, as they are unable to fly and must crawl on the treated surfaces, resulting in overexposure, not only through ingestion but also by inhalation. Scudeler et al. [32] also demonstrated the toxic effect of azadirachtin compounds by ingestion exposure in the third instar larvae of *C. claveri*.

These compounds induce antifeedant effects. A feeding antifeedant or deterrent is defined as a chemical that inhibits feeding without directly killing the insect; often, the insect remains near the treated plant and may eventually die from starvation [22]. Increase in mortality at the final larval stage could be explained by the antifeedant effect. The 0.3% and 0.5% doses of Azamax™ showed no lethal effects on *C. claveri* first and second instar larvae. Certain insects may ingest azadirachtin and not die immediately, they stop feeding shortly after and subsequently show signs of developmental toxicity [22]. Furthermore, the antifeedant activity of azadirachtin has also been reported in other insect species [22,39].

Despite this toxic effect on larvae of the third instar, this developmental stage showed less susceptibility than the prepupal and pupal stages. The significant alterations shown during larval–pupal development of *C. claveri*-treated insects demonstrate that Azamax™ mainly affects the prepupal and pupal stages. Deleterious metamorphic effects and a decrease in pupation seem to be associated with the interference of azadirachtin on the endocrine system [40]. These alterations related to the azadirachtin present in Azamax™ probably result in growth inhibition and molting defects in the insect [41,42]. This product is known to induce degenerative structural changes in the nuclei of all endocrine glands (prothoracic gland, corpus allatum, and corpus cardiacum) responsible for controlling molting and ecdysis in insects, which contributes to a generalized disruption of neuroendocrine function [39]. Bezzar-Bendjazia et al. [43] examined the lethal and sublethal effects of azadirachtin on *Drosophila melanogaster* and reported that the mortality at the immature stage increases directly proportionally to the azadirachtin doses and that the pupae represent the most affected stage, in agreement with our results.

In addition, Scudeler et al. [27,31] showed similar results related to the nonformation of pupae and an increase in the percentage of nonviable prepupa owing to the absence of cocoon formation when *C. claveri* larvae were exposed to different treatments of neem oil that contained approximately 1500 ppm azadirachtin as the active ingredient. These authors confirmed that azadirachtin interferes with molting and cocoon spinning, decreasing wall thickness and impairing attachment to a substrate. These negative impacts may reduce the effectiveness of the protective and mechanical functions of cocoons during pupation, which makes the insect more vulnerable to environmental factors [27].

Medina et al. [44] also reported toxicological effects in the pupal stage in green lacewing. These authors noted that azadirachtin was harmful at higher doses during the formation of the pupa when the predator *Chysoperla carnea* was exposed by topical application. Pupae of *Partamona helleri* queens that ingested an azadirachtin-contaminated diet during postembryonic development also presented offspring with malformations, and most of them were unable to walk or feed [35]. In contrast, Medina et al. [45] demonstrated the absence of alterations in young and old pupae of *C. carnea* after topical treatment with azadirachtin. They explain these results due to the silk ovoid cocoon that covers the decticous pupae, which might inhibit the uptake into the insect body after topical application. Nonetheless, they also found that azadirachtin significantly reduced the percentage of pupae when last instar *C. carnea* larvae were exposed by residual contact, indicating that susceptibility to azadirachtin varies with the application method and duration of the exposure, as reported by Zhang et al. [36].

Indeed, azadirachtin affects various biological processes, including larval and pupal development, by regulating the genes involved in insect growth [24]. In holometabolous insects, such as *C. claveri*, the pupal stage showed a drastic remodeling of most tissues and organs, which represents a critical phase to achieve adult formation [46] because this stage is a vulnerable part of the *C. claveri* lifecycle, and azadirachtin can induce several morphogenetic alterations in the pupa, preventing the complete emergence of the adult [40].

The molecular mechanisms of the sublethal effects of ingested azadirachtin are unclear, but in *D. melanogaster* larvae, investigations have demonstrated that azadirachtin mainly affects the regulation of posttranscriptional enzymes, proteins involved in cytoskeleton development, transcription, translation, regulation of hormones, and energy metabolism [24,47]. In addition, a recent study demonstrated that larval exposure to Azamax™ was able to induce DNA damage in the germline of female adults of *C. claveri* [33]. These authors also reported an increase in malformed adults in the Azamax™ treatments. In the present study, the observation of malformations in females was also observed, i.e., nonviable adults that did not survive. Malformations included insects with disrupted metamorphosis (with parts of the old exuvium remaining adhered to the adult body), lower size, contorted antennae, and abnormal legs and wings (Figure 1H,I and Figure 2). The literature reports that azadirachtin exerts similar effects in bee species [35,48] and grasshoppers [49]. In the case of *D. melanogaster*, azadirachtin has been shown to reduce lifespan [36]. Consistent with the findings of this study, Scudeler et al. [31] observed that all doses of neem oil had highly detrimental effects on the life cycle of the predator *C. claveri*.

In our experiments, although no dose-dependent relationship was observed with azadirachtin treatment, it did not affect the total life cycle; however, it did influence the development of each individual insect stage. Azadirachtin is reported to impair the development and survival of many different insect species. As demonstrated by Kraiss and Cullen [50], neem-derived insecticides affected the development time of *Aphis glycines*, an invasive pest insect, and its biological control agent, *Harmonia axyridis*. Scudeler et al. [31] demonstrated that neem oil induces an extension of the larval instars in *C. claveri* at all evaluated doses, and azadirachtin was found to cause significant developmental delays during the larva-to-pupa and pupa-to-adult transitions.

Lai et al. [24] previously reported a delay in pupation time in the third instar of *D. melanogaster* treated with azadirachtin and showed that azadirachtin causes downregulation of genes involved in hormonal regulation of development and probably induces the deleterious metamorphic effect observed in the present study. Similar to our results, Bernardes et al. [35] reported that azadirachtin treatment resulted in a reduced survival period, altered the development time, and caused morphological deformations. Moreover, Qiao et al. [51] reported that the neurotoxic effect of azadirachtin might interfere with different endocrinological and physiological processes in insects.

Exposure to Azamax™ induced morphological changes in the ovaries of female adults of *C. claveri* when compared with the control group (Figure 5 and Figure 6). External abnormalities in the reproductive system and significant alterations in the normal ovarian follicle were observed. Azadirachtin treatment impaired the development of the reproductive system, as recently reported by Bernardes et al. [35]. Several reproductive effects due to exposure to azadirachtin have been reported in many insects [31,32,33,35,43,49,52].

At both doses of Azamax™, the ovarioles of females treated with Azamax™ exhibited a number of protuberances resembling large pores on the ovariolar sheath. Subsequently, the sheath appeared thinner compared to that observed in the ovarioles of control females, as shown in Figure 5D. According to Cruickshank [38], the increase in large pores on the ovariolar sheath is likely associated with larger intercellular spaces between the cells comprising the ovarian follicles. These changes can be comparable to citronella oil, which induced damage to the ovarioles of *Spodoptera frugiperda* [53]. Oil-treated ovaries showed follicular cell stratification and removal and a thinner conjunctive sheath. Giorgi et al. [54] proposed that the enclosure and closeness exhibited by developing ovarioles through sheaths are designed to guarantee protection of the oocyte and to allow a directional flow of hemolymph through the insect ovary. Therefore, the external disorganization observed in the ovarioles of the insects exposed to Azamax™ can result in reduced reproduction.

Moreover, as observed in Figure 5B, ovarioles of treated insects at both doses presented a shrinkage represented by the larger terminal filament, and the vitellarium and germarium regions seemed to be smaller when compared to the control. These results agree with Ghazawi et al. [49], who showed that *Heteracris littoralis* treated with azadirachtin also suffered shrinkage of the ovary, associating this alteration with the interference of azadirachtin on the vitellogenesis process. Vitellogenesis is a complex process involving the deposition of yolk in the oocyte, resulting in a rapid increase in oocyte size [49]. Bernardes et al. [35] also described this effect on stingless bees, *Partamona helleri*, demonstrating a size reduction of the queens’ reproductive organs after treatment with an azadirachtin-based biopesticide.

Ovarian follicles of *C. claveri* treated with AzamaxTM did not complete their development (Figure 6A,E). Ghazawi et al. also reported inhibition of follicle development. The authors of [49] described that azadirachtin administered at a specific time can cause serious damage to oocytes. Medina et al. [52] pointed out that a commercial product based on azadirachtin (Align) interfered with the synthesis of vitellogenin and/or its uptake by oocytes. In this study, the developing follicles in the treated ovaries were significantly smaller than those in the control group.

Azadirachtin may inhibit vitellogenin synthesis and/or absorption, which eventually leads to the inhibition of both ovarian ecdysteroid synthesis and oogenesis processes, negatively impacting ovarian development, delaying vitellogenin synthesis, and finally absorption by ovarian follicles [40,55]. Lower levels of ovarian proteins, such as vitellogenin, suggest a reduced level of vitellogenesis, as reported in *Spodoptera exempta* [56] and *D. melanogaster* [57] treated with azadirachtin. This compound also prevents or alters the formation of new actin cytoskeleton proteins, resulting in the disruption of cell division and blocked transport, which may affect the process of dumping of the cytoplasmic contents from nurse cells to the oocyte [40]. Indeed, Anuradha et al. [58] reported that azadirachtin induces the depolymerization of actin in *D. melanogaster*, leading to cell cycle arrest and subsequent apoptosis.

Bernardes et al. [35] suggested that azadirachtin can impair the development of the insect reproductive system, which might be critical for the maintenance and reproduction of the species. The impact of azadirachtin on female reproduction could be explained by this interference of juvenile hormones and ecdysteroids [40], eventually leading to abnormal ovaries and an associated decrease in fecundity [40].

## 4. Materials and Methods

### 4.1. Chemicals

The biopesticide analyzed, available as an emulsifiable concentrate formulation, Azamax™ (active ingredient azadirachtin 12 g L^−1^; 1.2% m/m), was obtained from UPL do Brasil Indústria e Comércio de Insumos Agropecuários S.A., Ituverava, SP, Brazil [6].

### 4.2. Insect Maintenance

The newly hatched *Ceraeochrysa claveri* larvae (from 0 to 12 h old) used in these experiments were obtained from the mass colony maintained at the Laboratory of Insects in the Department of Structural and Functional Biology at the Institute of Biosciences of Botucatu at UNESP, Brazil. The *C. claveri* colony was reared with *Diatraea saccharalis* (Lepidoptera: Crambidae) eggs ad libitum during the larval stage and with an artificial diet for adults composed of 1:1 honey/yeast solution. The insect colonies and bioassays were maintained in an environmental chamber under controlled conditions: 25 ± 1 °C; 70 ± 10% RH; and a photoperiod of 12 h [31].

### 4.3. Bioassays

Two experimental concentrations of Azamax™, established at 36 mg/L (0.3%) and 60 mg/L (0.5%), were prepared separately with the addition of distilled water to obtain the desired concentrations. Fresh *D. saccharalis* egg clusters, recently deposited by females, were collected and dipped once in the tested doses of Azamax™ (0.3% and 0.5%), based on the recommended application rates for Brazilian agricultural fields. These doses correspond to the minimum and maximum field-recommended concentrations (MFRCs) as per the registered label rates of usage in agricultural fields where lacewings are present [34]. Eggs of each dose of treatment (diluted in distilled water to obtain the desired concentrations) were immersed for 5 s and air-dried at room temperature for 1 h. For the control group, egg clusters were dipped in distilled water [27,28,29,30,31,33,59,60].

Newly hatched larvae were selected randomly and placed in individual polyethylene cups of 2 cm height and 6 cm diameter to avoid cannibalism and to be chronically exposed to azadirachtin by ingestion. These were divided into three experimental groups. The groups were tested under the same environmental conditions as those described for rearing. In the control group (*n* = 150), larvae were fed ad libitum on *D. saccharalis* eggs treated with distilled water. In the experimental groups of Azamax™ at the 0.3% (*n* = 150) and 0.5% (*n* = 150) doses, larvae were fed ad libitum on eggs treated with the biopesticide throughout the larval period (15 days) until pupation. The egg clusters were replaced every 3 days to avoid their nonpredation by larvae and degradation of azadirachtin by ultraviolet light (adapted from Scudeler et al. [31]). After cocoon spinning by prepupae, specimens remained in the same polyethylene cups under the same controlled conditions for the entire experiment until the adults emerged (Figure 1A). Adults were kept in a polyethylene box of 9 cm height and 18 cm diameter and fed an artificial diet (1:1 honey/yeast solution) [33]. One day after they had emerged, the female insects were taken to obtain their ovaries for morphological analysis.

### 4.4. Toxicity Analysis

For larval instars, the cumulative mortality of the specimens was checked daily by monitoring larval mortality for fifteen days. Individuals that did not move after being touched with a fine brush were considered dead, according to Scudeler et al. [31]. A total of 450 larvae were sampled, with 150 individuals used per treatment (i.e., biopesticide dose, including the control). At the prepupal period, when the larva finished spinning cocoons, the eventual inability or failure of a larva to spin a cocoon to start the prepupal period was considered a dead prepupa (Figure 1B). At the pupal stage, in which larva remained inside the cocoon and transformed into an exarate pupa (Figure 1C), pupal mortality was considered when the pupa died inside of the cocoon (nonviable pupa) (Figure 1F). The percentages of larval mortality, pupae formed, and successful adult emergence from those pupae were recorded. Cumulative mortality was the percentage of total individuals who failed to reach the complete adult form.

Morphological changes in the prepupae, pupae and adults, cocoons spun by larvae in the prepupal period, viable and nonviable pupae, and malformed adults emerging from the larvae untreated and treated with Azamax™, were analyzed and documented with an Olympus SZX16 stereo microscope with cell D imaging software (Olympus Soft Imaging Solutions GmBH, Münster, Germany) [27].

### 4.5. Development Time

The developmental times of the larval, prepupal, and pupal stages were monitored daily. The total duration of the lifecycle was also recorded. The transition of larval instars was confirmed by the exuviae left after each ecdysis and by the color and external morphology of the larvae, as reported by Scudeler et al. [31].

### 4.6. Morphology of the Female Reproductive System

#### 4.6.1. Transmission Electron Microscopy (TEM)

For TEM, adult ovaries (*n* = 10 specimens per group) were collected 24 h after emergence. The specimens were quickly cryoanesthetized at −4 °C, and the ovaries were removed and fixed in 2.5% glutaraldehyde and 4% paraformaldehyde solution in 0.1 M phosphate buffer (pH 7.3) for 24 h at room temperature and then postfixed in 1% osmium tetroxide in the same buffer for 2 h. After washing in distilled water, the samples were contrasted with an aqueous solution of 0.5% uranyl acetate for 2 h at room temperature, dehydrated in a graded acetone series, and embedded in Araldite resin, as described previously by Santorum et al. [61]. Ultrathin sections were contrasted with uranyl acetate and lead citrate and then analyzed and photographed with a Tecnai Spirit transmission electron microscope (FEI Company, Eindhoven, The Netherlands) at the Electron Microscopy Center of the Institute of Biosciences of Botucatu.

#### 4.6.2. Scanning Electron Microscopy (SEM)

One of the ovaries of each pair (*n* = 10 specimens per group) was isolated and fixed in 2.5% glutaraldehyde in 0.1 M phosphate buffer (pH 7.3) for 48 h at room temperature. Thereafter, the samples were washed in distilled water, postfixed in 1% osmium tetroxide diluted in distilled water for 30 min at room temperature, dehydrated through a graded series of ethanol, critical point-dried with CO_2_, and coated with gold using a SCD 050 (Bal-Tec, Los Angeles, CA, USA), as described previously by Santorum et al. [61]. The samples were examined and photographed using an FEI Quanta 200 scanning electron microscope (FEI Company, Eindhoven, The Netherlands). The wings of *C. claveri* adults (control and experimental groups) were also analyzed and documented via SEM. Ten wing samples from the three experimental groups were collected and processed for SEM. The samples were desiccated in an oven at 25 °C overnight, mounted on stubs, and then coated with gold using a SCD 050 (Bal-Tec, Los Angeles, CA, USA). They were examined in an FEI Quanta 200 SEM with an accelerating voltage of 12.5 kV at the Electron Microscopy Center of the Bioscience Institute.

### 4.7. Statistical Analysis

Studies of the relative associations to the mortality of larvae, percentage of pupation and emergence, and cumulative mortality were conducted using the Chi-square test, which evaluated contrasts among and within multinomial populations. The tests were performed at a significance level of 5%.

To assess the developmental times of the larvae, prepupae, pupae, and total duration of the life cycle, data are presented as the mean ± standard error of the mean (X ± SEM). One-way ANOVA was used to evaluate mean differences between groups, previously checked for normality and variance homogeneity, using Kolmogorov–Smirnov and Bartlett tests, respectively. When normality was not achieved, the nonparametric Kruskal–Wallis test complemented with Dunn’s test was used instead. The criterion of significance was set at *p* < 0.05.

## 5. Conclusions

In conclusion, the results demonstrate that *C. claveri* is significantly impacted by the indirect ingestion of an azadirachtin-based biopesticide (Azamax™), causing mortality and various sublethal effects on fitness, development, and reproduction. Therefore, assessing the effects of natural compounds across all developmental stages of natural enemies is essential to ensure their safety and prevent potential negative impacts on *C. claveri* populations in agroecosystems, which could compromise effective pest control.

## Figures and Tables

**Table 1:** Effects on cumulative larval mortality (%) of *C. claveri* during larval exposure to Azamax™ using the maximum (0.5%) and minimum (0.3%) field recommended concentrations for agricultural crops (*n* = 150 larvae/group).

Treatment	First Instar *	Second Instar *	Third Instar *
Control	2.0 ^a^	5.4 ^a^	6.8 ^a^
Azamax™ (%)			
0.3	6.7 ^a^	13.1 ^a^	22.3 ^b^
0.5	6.0 ^a^	12.4 ^a^	21.5 ^b^

* Different letters within columns indicate significant differences (*p* < 0.05; chi square).

**Table 2:** Effects on the development and mortality of *C. claveri* after larval exposure to Azamax™ using the maximum and minimum field recommended concentrations for agricultural crops (*n* = 150 larvae/group).

Treatment	Pupation ^a^ (%) *	Lifecycle Time ^b^ (Days) **	Adult Emergence ^c^ (%) *	Cumulative Mortality ^d^ (%)*
Control	92.0 ^a^	27.7 ± 0.2 ^a^	92.0 ^a^	8.0 ^a^
Azamax™ (%)				
0.3	54.7 ^b^	28.5 ± 0.3 ^a^	51.3 ^b^	48.7 ^b^
0.5	40.0 ^c^	27.1 ± 0.3 ^a^	35.3 ^c^	64.7 ^c^

* Different letters within columns indicate significant differences (*p* < 0.05; chi square). ** Data (mean ± SE) followed by the same letter did not significantly differ (Kruskal–Wallis, *p* < 0.05) for adult emergence. ^a^ Percentage of formed pupae compared with the total number of treated larvae. ^b^ Number of total life cycle days per insect. ^c^ Percentage of emerged adults compared with the number of formed pupae. ^d^ Percentages of dead larvae, prepupae, pupae, and adults that failed to molt.

## Data Availability

Data are contained within the article. Further raw data will be made available by the authors upon request.
